# Measuring inefficiency for specific inputs using data envelopment analysis: evidence from construction industry in Spain and Portugal

**DOI:** 10.1007/s10100-017-0473-z

**Published:** 2017-04-12

**Authors:** Magdalena Kapelko

**Affiliations:** Department of Logistics, Institute of Applied Mathematics, Wroclaw University of Economics, Wrocław, Poland

**Keywords:** Data envelopment analysis, Input-specific inefficiency, Directional distance function, Construction industry

## Abstract

This article contributes to the efficiency literature by defining, in the context of the data envelopment analysis framework, the directional distance function approach for measuring both technical and scale inefficiencies with regard to the use of individual inputs. The input-specific technical and scale inefficiencies are then aggregated in order to calculate the overall inefficiency measures. Empirical application focuses on a large dataset of Spanish and Portuguese construction companies between 2002 and 2010 and accounts for three inputs: materials, labor and fixed assets. The results show, first, that for both Spanish and Portuguese construction companies, fixed assets are the most technically inefficient input. Second, the most inefficient scale concerns the utilization of material input in both samples; the reason for this inefficiency is that firms tend to operate in the increasing returns to scale portion of technology set. Third, in both samples, large firms have the lowest input-specific technical inefficiencies, but the highest input-specific scale inefficiencies, compared to their small and medium-sized counterparts, and tend to suffer from decreasing returns to scale. Finally, in both samples, input-specific technical inefficiency under constant returns to scale increased during the period of the recent financial crisis, mainly due to the augmentation in scale inefficiency.

## Introduction

The analysis of inefficiency with regard to inputs employed in the production process can provide valuable insights for firm managers looking to improve their use of these inputs, allowing for better decision making. Information on input-specific inefficiency levels is also useful for policy makers in terms of defining the policy instruments that promote greater levels of efficiency of firms’ inputs and estimating the effectiveness of policies that have already been implemented.

The measurement of inefficiency of decision making units (DMUs) with reference to the potential for input-specific reductions has attracted increasing interest in the recent data envelopment analysis (DEA) literature. The origin of input-specific inefficiency is related to the concept of input subvector technical efficiency introduced by Färe et al. (1994), which focuses on measuring the efficiency of firms relative to a subset of inputs rather than to the entire vector of inputs.^1^ In particular, subvector efficiency for a specific input indicates the possibility to contract this input, holding other inputs and outputs constant. Since Färe et al. (1994) developed the DEA radial measure of subvector efficiency, numerous studies have used this concept to analyze input-specific efficiency, mainly in the agribusiness sector. For example, Oude Lansink et al. (2002) analyzed the subvector efficiency in the sample of conventional and organic farms in Finland. Oude Lansink and Silva (2003b) and Oude Lansink and Bezlepkin (2003a) computed the efficiency measures with regard to $\hbox {CO}_{2}$ emissions and energy use, drawing on the data on specialized vegetable firms and greenhouse firms in the Netherlands, respectively. D’Haese et al. (2009) measured the efficiency in the use of land by dairy farms on the French Reunion Island.

Other studies of input-specific inefficiency in the DEA framework have explored the use of the measure known as the Russell or Färe–Lovell measure (Färe and Lovell 1978), which minimizes the arithmetic mean of the reductions in each input dimension, or related slack-based measure (Tone 2001), which maximizes all input and output slacks. Within this line, Oude Lansink and Ondersteijn (2006) used the multiplicative distance function to analyze the efficiency of energy use in Dutch greenhouse firms. More recently, DEA models based on the directional distance function have been considered for the measurement of input-specific inefficiency. These models are based on the directional slack-based measure proposed by Fukuyama and Weber (2009), which incorporated directional distance function technology into the slack-based measure. Examples of such models include that outlined by Skevas and Oude Lansink (2014), which provided evidence of inefficiency in the use of pesticides in Dutch arable farming, and the model put forth by Zhou et al. (2012), which estimated energy and $\hbox {CO}_{2}$-emission performance in electricity generation in OECD countries.^2^ However, all of these studies have focused exclusively on the input-specific technical inefficiency, neglecting the important component of input-specific scale inefficiency and adjustments in the scale of operation of each input that can provide important insights for overall efficiency improvements.^3^

The present study contributes to the DEA literature by developing a directional distance function approach for measuring both technical and scale inefficiencies with regard to the use of individual inputs, applying the directional slack-based measure suggested by Färe and Grosskopf (2010). An input-specific scale inefficiency measure is generated as the difference between input-specific technical inefficiency under constant returns to scale and input-specific technical inefficiency under variable returns to scale. The input-specific technical and scale inefficiencies are then aggregated to calculate the overall inefficiency measures.

Empirical application focuses on a large sample of Spanish and Portuguese construction firms between 2002 and 2010. In most countries, the expansion of economies is largely dependent on the construction sector, and Spanish and Portuguese construction industries are no exception. The construction industry is one of the largest sectors in both countries. In terms of value added, construction sector in 2007 represented an 11% and a 7% share of the total value added of all economic sectors in Spain and Portugal, respectively (Eurostat 2016a). These values considerably decreased due to the 2008 economic crisis to 6 and 4% in 2014 in Spain and Portugal, respectively. By 2014, construction output in Spain and Portugal experienced a loss of more than half the level prior to the crisis in 2007 (Eurostat 2016b). It is also worth noting that the average output loss in construction sector due to the crisis in all European Union (EU) countries was considerably lower, accounting for 20% (Eurostat 2016b). The Spanish and Portuguese construction firms were among the most adversely impacted of all EU countries by the economic crisis, mainly in terms of output loss, employment loss and decrease in construction permits (Eurostat 2016b; European Construction Industry Federation 2016). All this raises questions about the performance and efficiency of construction firms in Spain and Portugal, and about the impact of crisis on efficient performance of Spanish and Portuguese construction firms.

To the best of our knowledge, this study is the first to analyze the inefficiency with respect to the use of individual inputs for the construction industry. Previous studies of the construction sector have analyzed inefficiency measures for all inputs employed simultaneously, without distinguishing the contribution of each input to overall firms’ performance. Those relate to construction companies in Spain (Kapelko et al. 2014; Kapelko and Oude Lansink 2015a), Portugal (Horta et al. 2012), Iran (Wong et al. 2012), Canada (Pilateris and McCabe 2003), Greece (Tsolas 2011), China (Xue et al. 2008; Zheng et al. 2011), and South Korea (You and Zi 2007). Given that the construction sector plays a central role in the Spanish and Portuguese economies and has been severely impacted by the 2008 economic crisis, the study of input-specific inefficiency of construction companies in these countries can help firms define strategies for survival during the crisis and long-run efficiency improvements. In particular, the knowledge of inefficiency with respect to the capital employed by construction firms could be of interest, as it is widely known that the construction sector is heavily embodied in capital.

The remainder of this article proceeds as follows. Section 2 develops the framework for measuring input-specific technical and scale inefficiencies. Sections 3 and 4 focus on the empirical application of the article: Sect. 3 describes the dataset of Spanish and Portuguese construction firms and Sect. 4 presents the results of input-specific technical and scale inefficiencies. The article ends with Sect. 5, which offers some concluding comments.

## Data envelopment analysis framework for measuring input-specific inefficiency

In order to introduce the method used in this article, some notation must be defined. In this article, $j =(1,\ldots ,N)$ decision making units (DMUs) are considered and each use a vector of inputs $x = (x_{1},\ldots ,x_{M})\in \mathfrak {R}_{+}^{M}$ to produce a vector of outputs $y = (y_{1},\ldots ,y_{s})\in \mathfrak {R}_{+}^{s}$.

The inefficiency of each unit is assessed with reference to the production technology *T* that transforms inputs into outputs and is defined as:

1 $$\begin{aligned} T=\left\{ {(x,y):x\,can\,produce\,y} \right\} \end{aligned}$$

This technology satisfies the assumptions described in detail in Färe and Primont (1995). Specifically, inactivity is allowed, “free lunch” is not allowed, free disposability of inputs and outputs, and technology set is compact and convex.

Inefficiency of firms can be assessed in both output-orientation, which measures the potential of DMU for output augmentation, and input-orientation, which assesses the potential of DMU for input reduction. In this article we are interested in assessing the inefficiency for a subset of inputs; therefore, we focus on the input-oriented inefficiency model. In the directional distance function context, Färe and Grosskopf (2010) defined the slacks-based measure of inefficiency as:

2 $$\begin{aligned}&{\vec {D}}_T (x,y;g_x ,g_y ) \nonumber \\&\quad =\sup \left\{ \sum _{i=1}^M \beta _i +\sum _{r=1}^S \gamma _r :(x_1 -\beta _1 g_{x1} ,\ldots ,x_M -\beta _M g_{xM} ,y_1\right. \nonumber \\&\left. +\gamma _1 g_{y1},\ldots ,y_S +\gamma _S g_{yS} )\in T \right\} \end{aligned}$$

where $\beta _{i}$ and $\gamma _{r}$ represent, respectively, the ith input-specific and rth output-specific inefficiency scores, and $g_x =(g_{x1} ,\ldots ,g_{xM} )$ and $g_y =(g_{y1} ,\ldots ,g_{yS} )$ are directional vectors that contract inputs and expand outputs, respectively.

The model (2) is non-oriented and its input-oriented version can be defined by assuming $g_y =0$ as follows:

3 $$\begin{aligned} {\vec {D}}_T (x,y;g_x )=\sup \left\{ {\sum _{i=1}^M {\beta _i:(x_1 -\beta _1 g_{x1} ,\ldots ,x_M -\beta _M g_{xM} ,y)\in T} } \right\} \end{aligned}$$

In the above formulation, $\beta _{i}$ measures the degree of technical inefficiency of input *i*, and $g_x =(g_{x1} ,\ldots ,g_{xM})$ represents the component of the directional vector determining the direction in which input $x_i$ can be scaled. It is worth noting that Färe and Grosskopf’s (2010) original proposal was non-oriented, and that they set a directional vector as g = (1, 1) for inputs and outputs. In this paper we apply an input-oriented model, and the value of the directional vector is the actual quantity of inputs $(g_{xi}={x}_{i})$. In addition, Färe and Grosskopf’s (2010) proposal is not directly intended for the measurement of input-specific inefficiency. However, as with any slack-based measure, Färe and Grosskopf’s (2010) approach can be used for that purpose.

To compute input-specific technical inefficiency defined by (3), the following optimization problem needs to be solved using DEA for each observation “0”:

4 $$\begin{aligned}&{\vec {D}}_T (x,y;g_x ) \nonumber \\&\quad =\mathop {\max }\nolimits _{\beta _{i} ,\lambda _{j}} \left( \sum _{i=1}^M {\beta _i } \right) \nonumber \\&\quad \quad \quad \hbox {s.t.}\quad \sum _{j=1}^N {\lambda _j y_{rj} } \ge y_{r0},\quad r=1,\ldots ,S \nonumber \\&\quad \quad \quad \quad \quad \,\, \sum _{j=1}^N {\lambda _j x_{ij} } \le x_{i0} -\beta _i g_{xi} \quad i=1,\ldots ,M \end{aligned}$$

The above model assumes the constant returns to scale (CRS) technology, which makes it possible to compute the input-specific technical inefficiency relative to the CRS technology $( TIECRS _i )$. The model computes the values of ${\beta _{i}}$, which provide the largest feasible contraction of each input if a firm has to operate efficiently given the directional vectors specified relatively to the CRS technology. Hence, the model measures input-specific inefficiency scores for the *i*th input. It uses $\lambda _j$ as intensity weights to form the linear combinations of *n* observed DMUs. The model also assumes the strong disposability of inputs and outputs.

The input-specific inefficiency under the variable returns to scale (VRS) technology is computed by adding the restriction that the sum of the intensity weights is equal to 1:

5 $$\begin{aligned}&{\vec {D}}_T (x,y;g_x ) \nonumber \\&\quad =\mathop {\max }\nolimits _{\beta _i^{ VRS } ,\lambda _j } \left( \sum _{i=1}^M {\beta _i^{ VRS } } \right) \nonumber \\&\quad \quad \quad \hbox {s.t.}\quad \sum _{j=1}^N {\lambda _j y_{rj} } \ge y_{r0},\quad \quad r=1,\ldots ,S \nonumber \\&\quad \quad \quad \quad \quad \sum _{j=1}^N {\lambda _j x_{ij} } \le x_{i0} -\beta _i^{ VRS } g_{xi} \quad i=1,\ldots ,M \nonumber \\&\quad \quad \quad \quad \quad \sum _{j=1}^N {\lambda _j =1} \end{aligned}$$

The above model computes $\beta _{i}^{ VRS }$; that is, the degree of technical inefficiency for input *i* under the VRS technology $( TIEVRS _{i})$.^4^

Analogous to the inefficiency measures for all inputs simultaneously, when input-specific technical inefficiency under VRS differs from input-specific technical inefficiency under CRS, this reflects the presence of input-specific scale inefficiency; that is, the potential efficiency gains that can be obtained from the scale adjustments. Hence, it would be interesting to extend the analysis of input-specific technical inefficiency to include the scale inefficiency component, as proposed in this study. Input-specific scale inefficiency ($$ SIE _{i}$$) can be computed as:

6 $$\begin{aligned} SIE _{i} = TIECRS _{i} - TIEVRS _{i} =\beta _{i} -\beta _{i}^ VRS \end{aligned}$$

Input-specific scale inefficiency itself does not reveal anything about the nature of returns to scale; that is, if scale inefficiency is due to decreasing returns to scale (DRS) (firms being too big) or increasing returns to scale (IRS) (firms being too small). In order to determine this, an additional DEA program needs to be used; this is a similar program to (5), but instead of the last constraint, the constraint $$\sum \nolimits _{j=1}^{N} \lambda _{j}\le 1$$ is added. This model makes it possible to compute the input-specific technical inefficiency relative to the non-increasing returns to scale technology (NIRS), which we denote as $$ TIENIRS _{i}$$. DMU falls into the decreasing-returns section of the technology set for the *i*th input when $$ TIENIRS _{i}= TIEVRS _{i}$$, and exhibits increasing returns for the *i*th input when $$ TIENIRS _{i}\ne TIEVRS _{i}$$. Of course, firms have an optimal size for the *i*th input when they are scale-efficient for this input; that is, when $$ TIECRS _{i}= TIEVRS _{i}$$.

Finally, we can compute the overall inefficiency measures that account for all inputs. Overall technical inefficiency under CRS $( OTIECRS )$, overall technical inefficiency under VRS $( OTIEVRS )$, and overall scale inefficiency $( OSIE )$ can all be calculated by summing up the respective input-specific measures and dividing the sum by the number of inputs:

7 $$\begin{aligned} OTIECRS= & {} \frac{1}{M}\sum _{i=1}^M { TIECRS _i} \end{aligned}$$

8 $$\begin{aligned} OTIEVRS= & {} \frac{1}{M}\sum _{i=1}^M { TIEVRS _i } \end{aligned}$$

9 $$\begin{aligned} OSIE= & {} \frac{1}{M}\sum _{i=1}^M { SIE _i } \end{aligned}$$

Note that the Färe and Grosskopf’s (2010) measure used in this article is similar to Fukuyama and Weber’s (2009) directional slacks-based measure of technical inefficiency.^5^ This difference is that the latter used $\max \left( {\frac{1}{M}\sum \nolimits _{i=1}^M {\beta _i } } \right)$ as the objective function in the DEA model, and therefore used the average instead of the total, which Färe and Grosskopf (2010) used.^6^ Färe and Grosskopf’s (2010) measure is also related to the weighted additive model that was first introduced in Lovell and Pastor (1995). That model uses $\max \left( {\sum \nolimits _{i=1}^M {w_i s_i } }\right)$ as the objective function, where $s_{i}$ are input slacks and $w_{i}$ are weights representing the relative importance of unit inputs.^7^ Finally, Färe and Grosskopf’s (2010) measure can be also considered as a Russell-type measure (or Färe-Lovell measure) (Färe and Lovell 1978) in the directional distance function context.

## Data

The SABI (“Sistema de Análisis de Balances Ibéricos”) database from Bureau Van Dijk is a source of data of construction firms in Spain and Portugal. The homogeneity of the sample is obtained by analyzing the firms for which the main activity is construction of residential and non-residential buildings (NACE Rev. 2 code 4120).^8^ We focus on small, medium and large firms, based on the European Union’s definition of these categories of firm size (European Commission 2003). This definition is based on two variables—the enterprises’ annual turnover and the number of employees—and has been extensively used in the previous literature.^9^ Furthermore, only companies that were active between 2002 and 2010 are included in the sample. Then, we removed firms with missing information, which resulted in a sample of 5780 Spanish firms (22,690 observations) and 1020 Portuguese firms (3755 observations). Finally, because it is well known that the DEA method is very sensitive to the presence of outliers in the sample, we detected outliers by adapting outliers-detection rules from statistics for the specific context of efficiency analysis by DEA. The statistical rules are based on creating intervals for variables based on their means/medians and standard deviations. We adapted these rules by applying them for ratios of output over any of the inputs used in DEA. In particular, an observation was defined as an outlier if the ratio of output over any of the inputs was outside the intervals of the median plus or minus two standard deviations. This method has been extensively applied in past efficiency studies (e.g., Geylani and Stefanou 2013; Kapelko et al. 2014; Horta and Camanho 2015; Kapelko et al. 2015b).^10^ Application of this method resulted in a sample of 5706 and 965 firms in Spain and Portugal, respectively, that were operating between 2002 and 2010. In total, the dataset equated to 22,384 observations of Spanish companies and 3583 observations of Portuguese companies (unbalanced panels). In the Spanish sample there are 17,777 observations for small firms, 3702 for medium firms and 905 for large firms. The Portuguese sample consists of 2997 observations for small firms, 478 for medium firms, and 108 for large firms.

The DEA model uses accounting data and is defined based on three input variables (fixed assets, labor and materials) and one output variable (operating revenues).^11^ Such input–output configuration is consistent with previous studies of efficiency in the construction industry (You and Zi 2007; Kapelko and Oude Lansink 2015a). The book value of fixed assets is a proxy for capital, and is measured in millions of euros at constant prices from 2002, which were obtained by deflating the fixed assets by the industrial price index for capital goods. In general, capital is probably the most complicated input to measure, and there is no ideal method for its measurement. It is evident that the measure of capital should reflect the contribution of capital to production, and should take into account the consumption of capital, which constitutes a negative change in its value (Diewert 2008). In order to derive complete capital accounts, in addition to fixed assets, inventories should be taken into consideration (Diewert 2008). Unfortunately, in this study we do not have access to this type of data. In the absence of it, the book value of fixed assets is used as a proxy for capital. The book value of fixed assets comprises tangible and intangible assets and financial investments, net of depreciation. In this case, depreciation reflects the consumption of fixed capital, which is a negative change in the value of the fixed assets used in production. This proxy is extensively applied in many studies of the construction industry (see, e.g., Xue et al. 2008; You and Zi 2007; Kapelko and Oude Lansink 2015a). Labor was measured by employee costs, expressed in millions of euros at 2002 constant prices and obtained using the price index for labor costs for the construction industry. Materials used in the construction production are proxied by material costs measured in millions of euros, which were converted to 2002 constant prices by applying the price index for materials for residential buildings. Operating revenues are also expressed in millions of euros and deflated using the price index for residential buildings (2002 = 100). All of the price indices are supported by the Spanish and Portuguese statistical offices. The main descriptive statistics for input–output variables for Spanish and Portuguese construction firms per firm’s size are summarized in Table 1. The table indicates the considerable differences between firms in both Spanish and Portuguese samples, as indicated by the coefficient of variation for input and output variables.

**Table 1:** Descriptive statistics, 2002–2010, millions of euros (2002 = 100)

Variable	Small			Medium			Large		
	Mean	Std. dev.	CV	Mean	Std. dev.	CV	Mean	Std. dev.	CV
Spain									
Fixed assets	1.082	5.906	5.458	6.242	30.135	4.828	57.970	173.998	3.002
Labor	0.709	0.531	0.749	2.249	1.778	0.791	23.258	47.248	2.031
Materials	2.234	1.477	0.661	11.147	6.708	0.602	125.191	250.144	1.998
Operating revenues	3.598	1.652	0.459	16.823	8.301	0.493	186.346	363.063	1.948
Portugal									
Fixed assets	0.942	2.886	3.064	4.072	6.830	1.677	24.341	33.512	1.377
Labor	0.453	0.411	0.907	1.995	2.213	1.109	13.532	16.149	1.193
Materials	1.257	0.978	0.778	4.679	3.536	0.756	23.263	24.667	1.060
Operating revenues	3.670	1.687	0.460	17.753	8.987	0.506	113.001	104.350	0.923

*Std. dev.* standard deviation, *CV* coefficient of variation

## Results

The calculations of input-specific inefficiencies were undertaken for firms in each year, with the Spanish and Portuguese samples treated separately, because it is reasonable to assume that construction firms in each country have different technologies for converting inputs into outputs. In this way, we estimated managerial inefficiency as per Charnes et al. (1981); that is, inefficiency assessed in relation to the firm’s own group frontier of best practices (in this case, country-specific inefficiency). Our empirical results are divided into two parts: first, we compute overall and input-specific technical and scale inefficiencies of firms in the samples; second, we show the results of input-specific inefficiencies according to firm size. These analyses are conducted for the whole period, 2002–2010, and for sub-periods. Throughout the results, we tested the differences in overall and input-specific measures between Portugal and Spain, between sub-periods and between firms of different sizes using the test proposed by Simar and Zelenyuk (2006), which is referred to as the S–Z test. This test was derived from the nonparametric test of the equality of two densities developed by Li (1996).

### Overall and input-specific technical and scale inefficiencies

Table 2 summarizes the arithmetic averages of (managerial) input-specific technical inefficiencies computed under CRS and VRS technologies and the residual (managerial) input-specific scale inefficiency during 2002–2010 for Spanish and Portuguese construction companies. Furthermore, Table 2 shows the arithmetic averages for (managerial) overall CRS technical inefficiency, overall VRS technical inefficiency, and overall scale inefficiency. The number of efficient DMUs (that is, with inefficiency scores equal to 0) is reported in brackets in each case. Table 2 also summarizes the inefficiency measures for inefficient firms only. Details on the returns to scale—that is, the number of DMUs that exhibit DRS and IRS—are shown in Table 3.

**Table 2:** Input-specific technical and scale inefficiencies in construction industries in Spain and Portugal, 2002–2010

Input	Technical inefficiency CRS		Technical inefficiency VRS		Scale inefficiency	
	All	Inefficient	All	Inefficient	All	Inefficient
Spain						
Fixed assets	0.897 (236)	0.907	0.824 (558)	0.845	0.074 (197)	0.074
Labor	0.817 (270)	0.827	0.732 (674)	0.755	0.085 (231)	0.086
Materials	0.744 (494)	0.761	0.549 (1726)	0.595	0.195 (401)	0.199
Overall	0.819 (150)	0.825	0.702 (469)	0.717	0.118 (152)	0.119
Portugal						
Fixed assets	0.822 (142)	0.856	0.749 (309)	0.820	0.073 (137)	0.076
Labor	0.724 (169)	0.760	0.590 (399)	0.664	0.134 (157)	0.140
Materials	0.787 (137)	0.819	0.649 (363)	0.723	0.138 (134)	0.143
Overall	0.778 (111)	0.803	0.663 (285)	0.720	0.115 (112)	0.119
Significance (*P* values for S–Z test)						
Fixed assets	2.22e−16***	2.22e−16***	2.22e−16***	2.22e−16***	2.22e−16***	2.22e−16***
Labor	2.22e−16***	2.22e−16***	2.22e−16***	2.22e−16***	2.22e−16***	2.22e−16***
Materials	2.22e−16***	2.22e−16***	2.22e−16***	2.22e−16***	2.22e−16***	2.22e−16***
Overall	2.22e−16***	2.22e−16***	2.22e−16***	2.22e−16***	0.001***	2.22e−16***

$${}^{***}$$ Denotes statistically significant differences between Spain and Portugal at the critical 1% level. Values in brackets indicate the number of efficient DMUs

**Table 3:** Returns-to-scale characteristics in construction industries in Spain and Portugal, 2002–2010

Input	Number of observations for DRS	Number of observations for IRS
Spain		
Fixed assets	5574	16,613
Labor	5554	16,599
Materials	5437	16,546
Portugal		
Fixed assets	1305	2141
Labor	1288	2138
Materials	1305	2144

The total number of observations (that is DRS, IRS, and optimal scale) in Spain is 22,384, and in Portugal it is 3583

It can be seen from Table 2 that in the Spanish construction industry, for the whole period, the average country-specific CRS technical inefficiency was 89.7% for fixed assets, 81.7% for labor, and 74.4% for materials. These large values of inefficiency suggest that there is substantial scope for efficiency improvement in the utilization of the analyzed inputs. Among these inputs, fixed assets have the highest, while materials have the lowest, technical inefficiency. The highest technical inefficiency of fixed assets suggests that Spanish construction companies are wasting fixed assets such as buildings, equipment, and machinery; therefore, the companies should have incentives to manipulate their inputs of fixed assets. This suggests a capacity underutilization related to fixed assets. This can be a direct consequence of the crisis that, through its negative influence on demand, forced construction firms to decrease the amount of output produced, leaving the fixed inputs underutilized. The efforts of companies to improve the technical efficiency of fixed assets should be directed towards decreasing the amounts of these inputs or improved use of these inputs. This can be done, for example, by selling some of the fixed assets or directing part of fixed assets for renting. The decomposition of country-specific CRS inefficiency indicates that the country-specific VRS inefficiency differs from the country-specific CRS inefficiency to the largest extent in the case of input of materials. As a result, materials have the highest values of country-specific scale inefficiency among the analyzed inputs (19.5%), which indicates that an adjustment of the scale of operation for this input could improve materials efficiency by 19.5%. The direction in which the materials scale should be adjusted is reflected in Table 3, which shows that the overwhelming majority of Spanish construction firms exhibit IRS for materials; that is, they have the potential to scale up their activities related to materials. Labor exhibits a scale inefficiency of 8.5%, while the lowest scale inefficiency of 7.4% is found for fixed assets. Therefore, although the fixed assets of Spanish construction companies have the highest values of technical inefficiency, the firms have fewer problems adjusting their scale of operation for this input. Nevertheless, the results in Table 3 indicate that for both labor and fixed assets, the direction for improvement for the majority of firms would be to scale up utilization of these inputs as these firms exhibit IRS. In addition, the values of input-specific and overall technical inefficiencies for inefficient firms are relatively large and range from 0.595 to 0.907, suggesting the considerable difference between technically inefficient and efficient firms in the sample with regard to the utilization of specific inputs and in overall terms. However, the values of input-specific and overall scale inefficiencies for inefficient firms that range from 0.074 to 0.199 suggest that the differences in scale inefficiency between scale-efficient and scale-inefficient firms are rather small.

Like their Spanish counterparts, the fixed assets of Portuguese construction companies are the most technically inefficient input (for both the country-specific CRS and VRS technical inefficiency scores). However, in contrast to the Spanish case, labor is the most technically efficient input in the Portuguese industry. Country-specific scale inefficiency for all inputs for the Portuguese construction firms shows the same pattern as for the Spanish sample; that is, scale inefficiency is found to be the lowest for fixed assets, followed by labor, while materials exhibit the highest values of scale inefficiency. As for the Spanish sample, the reason for the input-specific scale inefficiency for the majority of Portuguese construction firms is IRS—that is, firms being too small—as shown by the data in Table 3. However, a considerable fraction of firms (less than 40% of the total sample) fall into the DRS group; that is, they should scale down their activities with regard to all inputs employed. As in the Spanish sample, the findings for Portuguese firms indicate a relatively large difference between technically efficient and inefficient firms, as well as a relatively small difference in scale inefficiency.

Several observations can be made when attempting to explain the differences in findings for input-specific inefficiencies between Spanish and Portuguese samples. Regarding inefficiency of fixed assets, construction firms in Spain are found to have higher inefficiencies than their Portuguese counterparts because the impact of crisis was more severe for Spanish firms (Eurostat 2016b); this could indicate a higher underutilization of fixed assets in the Spanish sample. Furthermore, in the aftermath of the crisis, southern European countries, including Spain and Portugal, initiated labor market reforms, implementing substantial deregulation regarding employment protection legislation (Eichhorst et al. 2016). The lower inefficiency of labor in the Portuguese construction industry than in the Spanish industry and the fact that labor is the most efficient input in Portugal seems to indicate that these reforms were more effective in Portugal. In fact, this seems to be confirmed by recent statistics showing that, for example, the increase in the share of temporary contracts and in the share of temporary contracts for workers aged 15–29 was considerably larger in Spain than in Portugal (Eichhorst et al. 2016). In addition, the lower inefficiency of materials in Spain’s construction industry than in Portugal’s, and the fact that materials are the most efficiently utilized input in Spain can be explained by, for example, the openings of construction innovation centers in Spain, which have aimed at, among other things, innovations in construction materials (European Commission 2016) that could render materials usage more efficient.

The high inefficiency levels found in this study might be due to the large sample sizes of Spanish and Portuguese construction firms, which consist of firms of very different sizes. In addition, in general, previous literature on construction industry efficiency has tended to report relatively large inefficiency values for this sector (see, e.g., Pilateris and McCabe 2003; Horta et al. 2012; Kapelko et al. 2014; Kapelko and Oude Lansink 2015a).

In order to provide insights into the temporal evolution of input-specific and overall inefficiency of Spanish and Portuguese construction firms, the samples were split into two sub-periods. The first sub-period encompasses the years 2002–2007, and concerns the pre-crisis period; the second sub-period consists of the years 2008–2010, and relates to the period of the recent financial crisis. Table 4 summarizes input-specific and overall inefficiency results for these sub-periods for the Spanish and Portuguese construction firms.

**Table 4:** Input-specific technical and scale inefficiencies in the construction industries in Spain and Portugal, for pre-crisis and crisis periods

Input	Technical inefficiency CRS	Technical inefficiency VRS	Scale inefficiency
Spain			
Pre-crisis (2002–2007)			
Fixed assets	0.895	0.835	0.060
Labor	0.806	0.734	0.073
Materials	0.694	0.507	0.187
Overall	0.798	0.692	0.106
Crisis (2008–2010)			
Fixed assets	0.905	0.790	0.115
Labor	0.847	0.727	0.120
Materials	0.896	0.676	0.220
Overall	0.883	0.731	0.152
Significance (*P* values for S–Z test)			
Fixed assets	2.22e−16***	2.22e−16***	2.22e−16***
Labor	2.22e−16***	2.22e−16***	2.22e−16***
Materials	2.22e−16***	2.22e−16***	2.22e−16***
Overall	2.22e−16***	2.22e−16***	2.22e−16***
Portugal			
Pre-crisis (2002–2007)			
Fixed assets	0.826	0.755	0.071
Labor	0.706	0.585	0.121
Materials	0.781	0.665	0.116
Overall	0.771	0.668	0.103
Crisis (2008–2010)			
Fixed assets	0.813	0.738	0.075
Labor	0.760	0.600	0.160
Materials	0.801	0.618	0.183
Overall	0.791	0.652	0.139
Significance (*P* values for S–Z test)			
Fixed assets	0.004***	2.22e−16***	2.22e−16***
Labor	2.22e−16***	0.401	2.22e−16***
Materials	0.001***	2.22e−16***	2.22e−16***
Overall	2.22e−16***	2.22e−16***	2.22e−16***

$${}^{***}$$ Denotes statistically significant differences between periods at the critical 1% level

By comparing the pre-crisis and crisis periods for Spanish construction firms, it can be seen that the average country-specific CRS technical inefficiency with regard to all inputs employed significantly increased during the period of crisis. This was mainly due to the significant increase in the average input-specific scale inefficiency. On average, country-specific VRS technical inefficiency for materials also increased during the crisis period compared to the pre-crisis period; however, it significantly decreased with regard to the inputs of fixed assets and labor. A comparison of the pre-crisis and crisis periods for the Portuguese construction firms in the sample reveals that, similarly to the Spanish sample, most of the country-specific inefficiency components increased during the crisis period. Therefore, the crisis may have had a negative impact on the Spanish and Portuguese construction firms’ performance. Only for CRS technical inefficiency for fixed assets and VRS technical inefficiency for fixed assets and materials did we find a significant decrease during the crisis period. Similarly to the Spanish firms, country-specific scale inefficiency significantly increased during the period of the financial crisis. Therefore, both Spanish and Portuguese construction firms faced more problems in finding the optimal scale of operations when the economic conditions worsened.

### Input-specific and overall technical and scale inefficiencies and firms’ size

Table 5 shows the (managerial) technical and scale inefficiency for materials, labor and fixed assets, as well as (managerial) overall measures for subsamples of Spanish and Portuguese construction firms, corresponding to three size classes: small, medium and large firms. The table also reports the number of efficient DMUs (in brackets). Data on the returns-to-scale parameters is given in Table 6.

**Table 5:** Input-specific technical and scale inefficiencies for size classes in construction industries in Spain and Portugal, 2002–2010

Input	Technical inefficiency CRS	Technical inefficiency VRS	Scale inefficiency
Spain			
Fixed assets			
Small	0.896 (182)	0.825 (320)	0.071 (149)
Medium	0.903 (39)	0.863 (99)	0.040 (35)
Large	0.901 (15)	0.652 (139)	0.250 (13)
Significance (S–Z test)	–	a, b, c	a, b, c
Labor			
Small	0.822 (213)	0.733 (405)	0.089 (175)
Medium	0.798 (43)	0.775 (115)	0.023 (42)
Large	0.790 (14)	0.540 (154)	0.250 (14)
Significance (S–Z test)	a, b, c	a, b, c	a, b, c
Materials			
Small	0.745 (382)	0.544 (1179)	0.201 (306)
Medium	0.738 (91)	0.628 (232)	0.110 (75)
Large	0.756 (21)	0.326 (315)	0.430 (20)
Significance (S–Z test)	–	a, b, c	a, b, c
Overall			
Small	0.821 (115)	0.701 (251)	0.120 (117)
Medium	0.813 (25)	0.755 (83)	0.057 (25)
Large	0.816 (10)	0.506 (135)	0.310 (10)
Significance (S–Z test)	a, b	a, b, c	a, b, c
Portugal			
Fixed assets			
Small	0.820 (123)	0.763 (196)	0.057 (119)
Medium	0.831 (15)	0.753 (57)	0.077 (14)
Large	0.839 (4)	0.351 (56)	0.488 (4)
Significance (S–Z test)	–	a, b, c	a, b, c
Labor			
Small	0.723 (143)	0.617 (263)	0.106 (131)
Medium	0.722 (22)	0.504 (76)	0.218 (22)
Large	0.755 (4)	0.210 (60)	0.545 (4)
Significance (S–Z test)	–	a, b, c	a, b, c
Materials			
Small	0.789 (119)	0.680 (229)	0.110 (116)
Medium	0.776 (15)	0.583 (66)	0.193 (15)
Large	0.783 (3)	0.098 (68)	0.685 (3)
Significance (S–Z test)	a, b, c	a, b, c	a, b, c
Overall			
Small	0.778(95)	0.687 (173)	0.091 (96)
Medium	0.776 (13)	0.614 (56)	0.163 (13)
Large	0.792 (3)	0.219 (56)	0.573 (3)
Significance (S–Z test)	a, b	a, b, c	a, b, c

Values in brackets indicate the number of efficient DMUs

${}^\mathrm{a}$ Denotes significant differences between small and medium firms at the critical 5% level

${}^\mathrm{b}$ Denotes significant differences between small and large firms at the critical 5% level

${}^\mathrm{c}$ Denotes significant differences between medium and large firms at the critical 5% level

**Table 6:** Returns-to-scale characteristics according to firm size in the construction industries in Spain and Portugal, 2002–2010

Input	Number for DRS	Number for IRS
Spain		
Fixed assets		
Small	3197	14,431
Medium	1582	2085
Large	795	97
Labor		
Small	3186	14,416
Medium	1574	2086
Large	794	97
Materials		
Small	3106	14,365
Medium	1543	2084
Large	788	97
Portugal		
Fixed assets		
Small	790	2088
Medium	411	53
Large	104	0
Labor		
Small	781	2085
Medium	403	53
Large	104	0
Materials		
Small	790	2091
Medium	410	53
Large	105	0

The total number of observations (that is DRS, IRS, and optimal scale) in Spain is 17,777 for small firms, 3702 for medium firms, and 905 for large firms, while the numbers of observations in Portugal are 2997, 478, and 108 observations for small, medium, and large firms, respectively

Table 5 shows that the findings regarding input-specific inefficiency observed for the entire sample of Spanish and Portuguese construction firms cannot necessarily be replicated for different size classes; that is, differences in inefficiency measures are observed between small, medium and large firms.

The results shown in Table 5 for the Spanish construction industry indicate that for the country-specific CRS inefficiency for the majority of measures, the differences between sizes are not statistically significant. On the other hand, significant differences between sizes are found for the country-specific VRS and scale inefficiencies, in terms of both overall measures and specific inputs. The findings regarding technical inefficiency under VRS show that large firms have the lowest values of inefficiency, while medium firms are the least efficient with regard to all inputs employed. This finding suggests that increasing size up to the largest size category may result in a decrease in inefficiency of use for all inputs. On the other hand, the opposite patterns are observed with regard to scale inefficiency: large firms have the highest values of scale inefficiency for all analyzed inputs, and medium firms have the lowest values. Hence, especially large firms in the sample of Spanish construction firms do not use their inputs at an optimal scale. The results in Table 6 show that the reason for this high scale inefficiency of large firms is that these firms tend to exist in the DRS portions of the technology set; that is, they are too big and should scale down their activities. For the Portuguese firms, the results in Table 5 show that the majority of differences between firm sizes are not statistically significant for country-specific CRS inefficiency. Therefore, with a few exceptions, the CRS inefficiencies, both in aggregate terms and for all inputs employed separately may be assumed to have the same distributions for small, medium and large firms. However, the country-specific VRS and scale inefficiencies for every input employed and in aggregate terms differ between sizes. Similarly to the Spanish construction firms, large Portuguese firms had the lowest values of technical inefficiency under VRS, and medium-sized firms had the greatest problems in terms of using their production potential, scoring the highest values of technical inefficiency. The findings regarding scale inefficiency for all inputs, and in overall terms, indicate that scale inefficiency increases with size; that is, large firms have the greatest difficulty combining their inputs to operate at the optimal scale, and small firms exhibited the lowest values for scale inefficiency. Therefore, the small Portuguese construction firms utilize their inputs at the most optimal scale. These findings suggest that large Portuguese construction firms are considerably larger than optimal scale. This is further confirmed by the results in Table 6, which reveal that almost all large Portuguese firms find decreasing returns to scale throughout the sample period, which suggests that the large firms in the sample exceeded the optimal scale. It is worth adding that for small Portuguese construction firms IRS were especially evident.

The input-specific and overall inefficiencies according to firm size for the pre-crisis and crisis periods are summarized in Table 7 (for Spanish firms) and Table 8 (for Portuguese firms).

**Table 7:** Input-specific technical and scale inefficiencies for size classes in the Spanish construction industry, for pre-crisis and crisis periods

Input	Technical inefficiency CRS	Technical inefficiency VRS	Scale inefficiency
Pre-crisis (2002–2007)			
Fixed assets			
Small	0.895	0.840	0.055
Medium	0.897	0.853	0.044
Large	0.896	0.656	0.240
Labor			
Small	0.810	0.734	0.076
Medium	0.792	0.775	0.017
Large	0.790	0.553	0.237
Materials			
Small	0.699	0.508	0.190
Medium	0.671	0.551	0.120
Large	0.685	0.296	0.390
Overall			
Small	0.801	0.694	0.107
Medium	0.787	0.726	0.060
Large	0.791	0.502	0.289
Crisis (2008–2010)			
Fixed assets			
Small	0.901	0.776	0.125
Medium	0.918	0.887	0.031
Large	0.913	0.643	0.271
Labor			
Small	0.860	0.776	0.130
Medium	0.812	0.774	0.038
Large	0.789	0.513	0.276
Materials			
Small	0.893	0.659	0.234
Medium	0.905	0.821	0.084
Large	0.906	0.391	0.515
Overall			
Small	0.885	0.722	0.163
Medium	0.879	0.828	0.051
Large	0.869	0.515	0.354
Significance (*P* values for S–Z test)			
Fixed assets			
Small	2.22e−16***	2.22e−16***	2.22e−16***
Medium	2.22e−16***	2.22e−16***	2.22e−16***
Large	2.22e−16***	0.133	0.490
Labor			
Small	2.22e−16***	2.22e−16***	2.22e−16***
Medium	2.22e−16***	2.22e−16***	2.22e−16***
Large	0.002***	0.070*	0.003***
Materials			
Small	2.22e−16***	2.22e−16***	2.22e−16***
Medium	2.22e−16***	2.22e−16***	2.22e−16***
Large	2.22e−16***	2.22e−16***	2.22e−16***
Overall			
Small	2.22e−16***	2.22e−16***	2.22e−16***
Medium	2.22e−16***	2.22e−16***	2.22e−16***
Large	2.22e−16***	0.071*	2.22e−16***

$${}^{***}$$ Denotes statistically significant differences between periods at the critical 1% level

${}^{*}$ Denotes statistically significant differences between periods at the critical 10% level

There was a significant improvement in the average country-specific VRS technical inefficiency for fixed assets during the crisis period, compared to the pre-crisis period, for the Spanish construction firms (see Table 4). The results according to size in Table 7 show that this improvement was due to small and large firms in the sample. Table 4 reported a general worsening of country-specific scale inefficiency during the crisis period. However, the results in Table 7 indicate that medium firms improved their scale inefficiency with regard to fixed assets and materials.

**Table 8:** Input-specific technical and scale inefficiencies for size classes in the Portuguese construction industry, for pre-crisis and crisis periods

Input	Technical inefficiency CRS	Technical inefficiency VRS	Scale inefficiency
Pre-crisis (2002–2007)			
Fixed assets			
Small	0.825	0.773	0.052
Medium	0.831	0.725	0.106
Large	0.836	0.353	0.483
Labor			
Small	0.706	0.620	0.086
Medium	0.702	0.450	0.251
Large	0.739	0.184	0.555
Materials			
Small	0.782	0.706	0.076
Medium	0.772	0.521	0.251
Large	0.774	0.093	0.680
Overall			
Small	0.771	0.700	0.072
Medium	0.768	0.565	0.203
Large	0.783	0.210	0.573
Crisis (2008–2010)			
Fixed assets			
Small	0.809	0.742	0.066
Medium	0.830	0.809	0.021
Large	0.845	0.348	0.497
Labor			
Small	0.759	0.613	0.146
Medium	0.763	0.611	0.152
Large	0.782	0.251	0.531
Materials			
Small	0.804	0.626	0.178
Medium	0.783	0.705	0.078
Large	0.798	0.105	0.693
Overall			
Small	0.791	0.660	0.130
Medium	0.792	0.708	0.084
Large	0.808	0.235	0.573
Significance (*P* values for S–Z test)			
Fixed assets			
Small	0.004***	2.22e−16***	2.22e−16***
Medium	0.400	0.004***	2.22e−16***
Large	0.323	0.353	0.785
Labor			
Small	2.22e−16***	0.237	2.22e−16***
Medium	2.22e−16***	2.22e−16***	2.22e−16***
Large	0.007***	0.295	0.310
Materials			
Small	0.003***	2.22e−16***	2.22e−16***
Medium	0.208	2.22e−16***	2.22e−16***
Large	0.407	0.162	0.172
Overall			
Small	2.22e−16***	2.22e−16***	2.22e−16***
Medium	0.024**	2.22e−16***	2.22e−16***
Large	0.315	0.141	0.954

$${}^{***}$$ Denotes statistically significant differences between periods at the critical 1% level

$${}^{**}$$ Denotes statistically significant differences between periods at the critical 5% level

While Table 4 showed that country-specific CRS and VRS technical inefficiencies for fixed assets in Portugal decreased during the crisis period compared to the pre-crisis period, Table 8 indicates that it was small firms that made these significant improvements in inefficiencies. In addition, the small firms improved their materials VRS inefficiency during the period of financial crisis. Furthermore, despite the general increase in country-specific scale inefficiency shown in Table 4, Table 8 indicates that medium firms managed to improve their scale inefficiency during the crisis period. In general, the results in Table 8 indicate that large Portuguese construction firms seem to have been the most adversely impacted by the crisis, although many of the differences between the pre-crisis and crisis periods for these firms were proven not to be statistically significant.

## Conclusions

The conventional approach in DEA is to analyze the inefficiency of decision making units with regard to all inputs employed in the production process. However, some inputs can be fixed or uncontrollable, so it might only be possible for firms to influence some of the inputs. Therefore, it would be useful to know the inefficiency of each input separately.

This study has developed a directional distance function approach for measuring input-specific technical and scale inefficiencies. The article uses DEA to decompose input-specific technical inefficiency under constant returns to scale into input-specific technical inefficiency under variable returns to scale and input-specific scale inefficiency. The empirical application focuses on the samples of Spanish and Portuguese construction firms engaged in the construction of residential and non-residential buildings between 2002 and 2010.

The results of the empirical application show the considerable technical inefficiencies of all inputs employed for both Spanish and Portuguese construction firms, which implies that there is substantial scope for efficiency improvement in the utilization of inputs. The findings clearly indicate that the firms in both the Spanish and Portuguese samples find it difficult to manage fixed assets exhibiting the highest technical inefficiency values for this input. Therefore, the maximum inefficiency comes from the inefficient use of fixed assets, which is the major input for construction firms operations. Better management of fixed assets by construction firms could help reduce technical inefficiency of this input. Applying existing technology that could include improvements in management and learning-by-doing of fixed assets by construction firms could help reduce the technical inefficiency of this input. Government policies could encourage incremental increases in the efficiency of fixed assets by encouraging firms to reduce the underutilization of fixed assets; for example, in the forms of preferential credits for possible buyers or activation of rental markets. This result could also imply that the amount of fixed assets that the construction firms in the samples possess is too high for the amount of output produced, which causes them to underutilize existing capacities with regard to fixed assets. It is possible that, in times of crisis that severely impacted both Spanish and Portuguese construction industry, the firms were forced to considerably reduce their activity, decreasing the output, but could not adjust their fixed assets levels instantaneously. On the other hand, Spanish and Portuguese construction firms have fewer problems managing their material and labor inputs; we found that the most technically efficient input is materials in Spain and labor in Portugal.

The results regarding input-specific scale inefficiency indicate that both Spanish and Portuguese construction firms operate on the least efficient scale with regard to materials, compared to other inputs employed. Hence, the adjustment of scale for materials provides substantial scope for efficiency improvement, which, for the majority of Spanish and Portuguese firms, should concern scaling up their activities with regard to materials. Furthermore, construction firms need more flexibility in adjusting their scale of operations for this input. Construction managers could rationalize the process of acquisition and use of materials on the basis of these findings. Policy makers could, for example, encourage firms to use innovative construction materials and/or undertake R&D activities with regard to construction materials.

This study also provides evidence regarding the differences in input-specific inefficiencies when controlling for firm size. In particular, our findings imply that large firms in both Spain and Portugal have the lowest input-specific technical inefficiencies, but the highest input-specific scale inefficiencies, compared to small and medium-sized firms. Hence, large firms manage their resources well, but do not choose an appropriate scale for the operation of their inputs. In particular, the majority of large firms exist in the DRS portion of the technology set, which indicates that the direction of improvement of their scale inefficiency lies in scaling down activities related to specific inputs.

This study analyzed the 2002–2010 period, which includes the 2008 financial crisis; hence, it also provides some insights into whether the worsening economic conditions impacted the construction firms’ input-specific inefficiencies. The comparison of average inefficiencies between the pre-crisis and crisis periods showed that, in general, in the construction industries in both Spain and Portugal CRS technical inefficiency significantly increased during the crisis, mainly due to the increase in scale inefficiency. However, the increase in scale inefficiency was not experienced equally by firms of all size categories, and medium firms in both Spain and Portugal managed to improve their scale inefficiency during the financial crisis.

This study analyzed input-specific inefficiency with regard to two dimensions—technical and scale—and further research may be carried out to explore measures of cost and allocative inefficiencies. Another interesting line for future investigations into input-specific inefficiency measurement would be to consider modeling fixed assets as a quasi-fixed input; for example, by developing a dynamic input-specific approach along the lines proposed by Silva and Stefanou (2003). Because the data used in this study made it impossible to classify firms into more detailed construction sectors—that is, to divide the sample into firms engaged in construction of residential buildings and those constructing non-residential buildings—future research study could focus on more detailed sectors of construction to identify whether differences in inefficiency exist between sectors. Another follow-up line of study could be to consider other proxies for capital of construction firms; for example, to distinguish fixed assets and the stock of unsold buildings (for the sample at hand, this would require the collection of additional data). Finally, a useful extension would be to develop input-specific inefficiency measures that account for the sampling noise; for example, through implementing the bootstrap method and analyzing the robustness of the study’s findings based on sampling.

## Footnotes

1 Earlier attempts to analyze inefficiency with regard to the use of specific inputs include the work of Banker et al. (1989) that use the concept of inefficiency of individual input as the amount of excess input plus the slack. This idea was recently applied in the study of Chen et al. (2005).

2 Another research line regarding input-specific inefficiency in the DEA framework applied the concept of multi-directional efficiency (Bogetoft and Hougaard 1999; Asmild et al. 2003), with the studies undertaken to analyze, for example, resource utilization in Danish elderly care (Hougaard et al. 2004), efficiency patterns in Chinese banks (Asmild and Matthews 2012) or efficiency of agricultural farms in Lithuania (Baležentis and Witte 2015; Asmild et al. 2016). Recently, multi-directional efficiency analysis was extended to dynamic context by Kapelko and Oude Lansink (2017) with application to the measurement of input-specific dynamic inefficiency of European dairy manufacturing firms.

3 There is also an important stream of research that analyzes the input-specific productivity evolution over time; this includes Mahlberg and Sahoo (2011), Chang et al. (2012) and Kapelko et al. (2015b). However, as the present study focuses on the input-specific inefficiency analysis in the specific point in time, those papers are not reviewed. Note that papers of Oude Lansink and Ondersteijn (2006) and Skevas and Oude Lansink (2014), in addition to analyzing input-specific inefficiency, also apply the input-specific productivity growth measures.

4 It is worth pointing out that there exists possibility of both models (4) and (5) to have multiple solutions. That is depending on the software used to compute efficiency one can obtain different solutions, which as a consequence might influence the results of scale inefficiency as well as returns to scale. However, this problem pertains to all previous studies of input-specific inefficiency and productivity growth and the solution to this problem does not exist in the context of the measurement of input-specific inefficiency and productivity growth. Therefore, this fact should be considered as a limitation of this study that opens up future research possibilities.

5 Moreover, it is easy to show that by using a specific directional vector Fukuyama and Weber’s (2009) measure can be reduced to Färe and Grosskopf’s (2010) measure. Therefore, the latter measure should be considered as a special case of the former measure.

6 Of course, $\beta _{i}$ will the same in both Fukuyama and Weber (2009) and Färe and Grosskopf (2010).

7 In particular, Pastor and Aparicio (2010) showed that applying some specific weights to the directional slacks-based measure of Fukuyama and Weber (2009), it becomes equivalent to the weighted additive model. However, Fukuyama and Weber’s (2009) measure was developed for purposes of: (1) studying Pareto-Koopmans efficiency in the directional model framework by taking into account nonzero slacks, (2) interpreting weights as directions in the directional model, and (3) showing relationships among existing efficiency/inefficiency measures, given a common directional vector. Therefore, these purposes are different than these of the weighted additive model.

8 NACE Rev. 2 is the classification of economic activities used by the European Union. In this classification, NACE Rev. 2 code 4120 is the most detailed distinction that can be achieved. Therefore, it was not possible to divide the sample further into construction of residential buildings and construction of non-residential buildings.

9 The alternative methods used in the literature for the purpose of firm-size measurement are usually based on one variable only; for example, they distinguish size classes based on total assets, or number of employees, or sales.

10 The use of this method identified similar outliers to the application of Simar’s (2003) procedure. Alternative methods of outlier detection are described in, for example, Wilson (1993), Seaver and Triantis (1995), and Fox et al. (2004).

11 It is worth noting that in the construction industry in general there exists a time lag between cost expenditures and the positive cash flows obtained from sales. The use of accounting data as a measure of inputs and outputs accounts for this possible time lag. The accounting data is prepared based on the principle of matching expenses to sales. In other words, the revenues for a period are matched with the expenses incurred in generating them; stock unsold at the end of the year did not contribute to the revenues of this year; therefore, it is carried forward in the balance sheet to be matched with revenues in the subsequent year. In particular, the data on revenues used in this study as an output includes an adjustment of the variation of stocks of finished goods and in the process of manufacture.
